# 144. Gram Negative Bacteria with Increased Minimal Inhibitory Concentration for Cefiderocol among Pediatric Patients in a Northern Italy Hospital.

**DOI:** 10.1093/ofid/ofac492.222

**Published:** 2022-12-15

**Authors:** Chiara Russo, Claudia Sette, Chiara Medici, Giuseppe Losurdo, Candida Palmero, Carolina Saffioti, Marcello Mariani, Erica Ricci, Elisabetta Ugolotti, Elisabetta Bondi, Shana Montalto, Elisa Tavella, Elio Castagnola, Alessio Mesini

**Affiliations:** IRCCS Policlinic San Martino, Genoa, Liguria, Italy; Istituto Giannina Gaslini, Genoa, Liguria, Italy; Istituto Giannina Gaslini, Genoa, Liguria, Italy; Istituto Giannina Gaslini, Genoa, Liguria, Italy; Istituto Giannina Gaslini, Genoa, Liguria, Italy; Istituto Giannina Gaslini, Genoa, Liguria, Italy; Istituto Giannina Gaslini, Genoa, Liguria, Italy; Istituto Giannina Gaslini, Genoa, Liguria, Italy; Istituto Giannina Gaslini, Genoa, Liguria, Italy; Istituto Giannina Gaslini, Genoa, Liguria, Italy; Istituto Giannina Gaslini, Genoa, Liguria, Italy; Istituto Giannina Gaslini, Genoa, Liguria, Italy; Istituto Giannina Gaslini, Genoa, Liguria, Italy; Istituto Giannina Gaslini, Genoa, Liguria, Italy

## Abstract

**Background:**

Cefiderocol is a siderophore cephalosporin active against Gram negative (GN) carbapenem resistant bacteria, approved in Italy in September 2020 for adult patients. In pediatric population off label use is allowed when severe infections from multidrug resistance (MDR) GN bacteria occur. Recently, some authors reported the emergence, among strains of *Enterobacterales* resistant to ceftazidime avibactam, of cross-resistance with cefiderocol.

The aim of this study is to describe GN strains with increased Minimal Inhibitory Concentration (MIC) for cefiderocol isolated in our hospital.

**Methods:**

This is a retrospective, single-center study conducted in Istituto Giannina Gaslini Pediatric Hospital (Genoa, Italy) from 1^st^ January 2020 to 31^st^ April 2022. We collected all strains of MDR GN bacteria whit increased MIC for cefiderocol isolated in our hospital. Cefiderocol susceptibility testing was performed by disk diffusion assay with the disk of 30 μg (Kirby-Bauer method, KB) according to EUCAST’s recommendations and KB breakpoints. We collected demographical data, previous colonization (if note), carbapenemases production, clinical outcomes (infections from MDR; death at 7 and 30 days; ICU admission).

**Results:**

Overall, 10 GN strains with increased MIC for cefiderocol were collected. 9/10 were *Enterobacterales* (4 *Escherichia coli*, 2 *Enterobacter cloacae complex*, 3 *Klebsiella pneumoniae*), one was *Acinetobacter baumannii*. 8/10 patients were admitted to other hospital in the six months before admission in our hospital, data about previous colonization were unknown. All MDR isolated produced metallo-β-lactamase [MBL] (5/10 New Delhi metallo-β-lactamase [NDM], 5/10 Verona integron-encoded metallo β-lactamase [VIM]). Four patients developed infection due to MDR (2 urinary tract infections, 1 bloodstream infection, 1 surgical wound), 2/4 patients died during the hospitalization.

**Conclusion:**

Our study confirms that MDR producing MBL carbapenemases may have reduce susceptibility to cefiderocol, even if never exposed to this siderophore cephalosporin.

Due to the possibility of cross resistance with MDR resistant to ceftazidime/avibactam, cefiderocol should be carefully prescribe in empirical treatment of MBL producers strains.

**Disclosures:**

**All Authors**: No reported disclosures.

